# Phenolic Compounds from Tropea Red Onion as Dietary Agents for Protection against Heavy Metals Toxicity

**DOI:** 10.3390/life14040495

**Published:** 2024-04-11

**Authors:** Rosanna Mallamaci, Filomena Conforti, Giancarlo Statti, Pinarosa Avato, Alexia Barbarossa, Daniela Meleleo

**Affiliations:** 1 Department of Biosciences, Biotechnologies and Environment, University of Bari “Aldo Moro”, 70125 Bari, Italy; 2 Department of Pharmacy, Health and Nutritional Sciences, University of Calabria-DFSSN, 87036 Rende, Italy; giancarlo.statti@unical.it; 3 Department of Pharmacy-Drug Sciences, University of Bari “Aldo Moro”, 70125 Bari, Italy; pinaavato@gmail.com (P.A.); alexia.barbarossa@uniba.it (A.B.); 4 Department of Science of Agriculture, Food, Natural Resources and Engineering, University of Foggia, 71122 Foggia, Italy; daniela.meleleo@unifg.it

**Keywords:** cyanidin, cyanidin-3-*O*-glucoside, heavy metals, quercetin, *Allium cepa* var. Tropea, Caco-2 cells

## Abstract

The present study aims to highlight the cell protective effect of Tropea red onion (TRO) hydroalcoholic extract and some of its components against “non-essential” heavy metals. For this purpose, the cytoprotective roles of cyanidin, cyanidin-3-*O*-glucoside and quercetin against Cd, Hg and Pb and of TRO extract against Hg and Pb have been investigated, and data are reported here. To the best of our knowledge, this is the first detailed evaluation of the protective effect against cell damage induced by “non-essential” heavy metals through the simultaneous administration of cyanidin, cyanidin-3-*O*-glucoside and quercetin with CdCl_2_, HgCl_2_ or PbCl_2_ and the TRO extract against HgCl_2_ and PbCl_2_. Present data are also compared with our previous results from the TRO extract against Cd. The antioxidant capacity of the extract was also determined by the ferric reducing antioxidant power (FRAP) and the bovine brain peroxidation assay. Both of the assays indicated a good antioxidant capacity of the extract. Cell viability and the impact on necrotic cell death were examined by the MTT (3-(4,5-dimethylthiazol-2-yl)-2,5-diphenyltetrazolium bromide) test and lactate dehydrogenase (LDH) release assay. After 24 h of exposure, Caco-2 cell viability decreased by approximately 50% at 0.25 μM for Cd, Hg and Pb and, after 72 h, the ranking order of “non-essential” heavy metal toxicity on cell viability was PbCl_2_ > CdCl_2_ > HgCl_2_. Cell viability was assessed by treating the cells with the biomolecules at doses of 25, 50 and 100 µg/mL for 24 and 72 h. The same analysis was carried out on Caco-2 cells treated with combinations of TRO extract, cyanidin, cyanidin-3-*O*-glucoside, or quercetin and “non-essential” heavy metals. Treatments with the bioactive metabolites did not significantly improve cell viability. The identical treatment of Caco-2 cells produced instead LDH release, suggesting a decrease in cell viability. Consistently with the finding that TRO extract showed a good antioxidant activity, we suggest that its higher cytotoxicity, compared to that of the individual assayed phytochemicals, may be derived by the combined antioxidant and chelating properties of all the molecules present in the extract. Therefore, from all the acquired experimental evidence, it appears that the TRO extract may be a better promising protective agent against the toxic effect of Cd, Hg and Pb compared to its bioactive metabolites.

## 1. Introduction

The term heavy metal(loid) defines chemical elements, characterized by metallic properties, that are often associated with pollution and the risk of biological toxicity for plants, animals, humans and for the environment [1]. Among them, there are metals like arsenic (As), cadmium (Cd), lead (Pb), mercury (Hg) and chromium (Cr) that are defined as “non-essential” and have no known biological function [2]. Conversely, heavy metals such as iron (Fe), zinc (Zn) and copper (Cu) that participate in metabolic processes are defined as “essential”. Excessive levels of both “essential” and “non-essential” heavy metals lead to toxic effects and cell death. “Non-essential” heavy metals usually enter the body by inhalation (industrial and urban fumes) or by ingestion (contaminated food and water), even if their absorption in the respiratory and digestive tracts varies from individual to individual depending on age and nutritional status [3]. Humans can also come into contact with “non-essential” heavy metals through daily use of some household items; mercury, for example, is contained in some disinfectants; cadmium is bound to nickel in batteries; lead is used in the coatings of mirrors, batteries and tiles [4,5].

Lead is primarily absorbed through the respiratory and digestive systems, but it can also enter the body through the skin. Exposure to this metal is linked to numerous nourishments and can affect the body’s neurological, biochemical and cognitive functions [6]. It may exert toxicity both by oxidative stress (the affinity for sulfhydryl groups also affects GSH, leading to imbalances between antioxidant systems and formation of ROS) [7,8] and by interference with the physiological ion fluxes (it can replace divalent cations such as Ca^2+^, Mg^2+^ and Fe^2+^, as well as Na^+^).

Poisoning by “non-essential” heavy metals is the consequence of their interference with the biochemical processes of the organism; when they are ingested, in the acidic environment of the stomach, they are converted into the more stable oxidation state (Cd, Hg, Pb) by binding to proteins and enzymes and then forming inactive adducts [9], or they can replace an enzymatic cofactor by altering its physiological functioning (for example, Cd^2+^ can replace Zn^2+^). A possible therapeutic approach in acute “non-essential” heavy metals poisoning is based on the use of chelation therapy to promote metal excretion. Dimercaprol (2,3-dimercaptopropanol), sodium calcium edetate and succimer (dimercaptosuccinic acid) are the three agents primarily used for chelation therapy. Once chelated, the metal is more easily eliminated from the body. However, this therapy is not without potential lethal complications [10]. Dietary supplements can be an advantageous alternative due to the fact that they can affordably and readily be included in the daily diet and contribute to reducing the adverse effects of the chelation effect and alleviating or preventing “non-essential” heavy metals toxicity [11]. In recent years, a large part of the scientific community has turned to the study and characterization of bioactive compounds deriving from numerous natural sources [12] since the numerous benefits they bring to human health are evident. Phytochemicals are often bioavailable, easily absorbed from the intestine and transferred into the circulation to reach the systems where they carry out their beneficial activity. However, individual pure bioactive molecules may lose their bioactivity or behave differently from when they are ingested as a combination in a complex natural extract [13].

Plants are major dietary sources of bioactive specialized metabolites, vital vitamins and metals according to a large number of studies. Some edible plants, such as tomatoes, berries, onions, garlic and grapes, are of particular importance as natural antagonists of “non-essential” heavy metals toxicity and should be consumed regularly [14]. Epidemiological studies showed that diets rich in vegetables and fruits can lower the risk of chronic diseases including cancer [15]. Polyphenols such as flavonoids, and anthocyanins, or simple phenolics from plant sources are the bioactive compounds responsible for these effects. They are strong antioxidants able to eliminate free radicals [16], inhibit or activate enzymes and act as metal chelators, thus preventing cellular proteins, lipids and nucleic acids damage [17]. Natural polyphenols also reduce leukocyte immobilization, can inhibit cell proliferation and angiogenesis, induce apoptosis and can exhibit phytoestrogen activity [14]. Flavonoids are highly antioxidant polyphenolic compounds with a protective role against heart disease, cancer, cognitive decline, diabetes and obesity [15,16]. Quercetin, for example, has a marked protective effect on Cd-induced toxicity, and it is a potent oxygen free-radical scavenger and a metal chelator [18]. It has been observed that anthocyanins extracted from blueberries have protective effects against Cd-induced liver toxicity in mice due to their anti-inflammatory and antioxidant effects [19,20]. Previous studies have pointed out that cyanidin-3-*O*-glucoside can reduce oxidative damage through free-radical scavenging and regulation of reductase activity [21]. Li et al., 2017 [22] showed that anthocyanins possessing ortho-dihydroxy groups on the B ring can bind “non-essential” heavy metals and thus reduce their concentration and their toxicity. Plant extracts can contain a very high number of different metabolites with variation in their occurrences and abundances, and it is generally accepted that the medical use of plant complex extracts of bioactive metabolites might be more effective than that of purified bioactive phytochemicals due to the beneficial synergic interactions [23,24]. The synergistic interactions between the constituents of a plant extract are a vital part of their medical application and therapeutic efficacy; although, crude plant extracts have been sometimes shown to have greater in vitro and/or in vivo bioactivity than isolated phytochemicals at an equivalent dose [25].

*Allium cepa* var. Tropea (Tropea red onion) is a unique cultivar grown in southern Italy (Calabria region) [26], and it is one of the richest natural sources of bioactive compounds such as polyphenols (flavonoids and anthocyanins), organosulfur components, inulin oligofructans and saponins, which have been shown to have biological and pharmacological roles both in in vitro and in vivo systems [27,28]. Thus, our earlier investigation of Tropea red onion (TRO) allowed us to demonstrate that the hydroalcoholic extract of the dry outer scales, containing quercetin, some quercetin glucosides and cyanidin-3-*O*-glucoside, has a good in vitro inhibitory activity against pancreatic lipase with potential as an anti-obesity agent [29,30]. In addition, it has been preliminarily established [31] that this extract exhibits a concentration- and time-dependent cytoprotective effect against Cd-induced injury in Caco-2 cells, an in vitro model for the intestinal barrier.

Based on the above-described results and as a further contribution to highlight the cell protective effect against “non-essential” heavy metals of TRO hydroalcoholic extract and some of its components, the present study aims to investigate the cytoprotective role of cyanidin and its glycosylated derivative cyanidin-3-*O*-glucoside and quercetin against Cd, Hg and Pb and of TRO extract against Hg and Pb. To the best of our knowledge, this is the first detailed evaluation of the protective effect against the toxicity damage induced by “non-essential” heavy metals through the simultaneous administration of cyanidin, cyanidin-3-*O*-glucoside and quercetin with CdCl_2_, HgCl_2_, or PbCl_2_ and of dry outer scales TRO extract against HgCl_2_ and PbCl_2_. Present data are also discussed and compared with previous results [31] from Tropea red onion extract against Cd.

As in our previous investigation, the human Caco-2 adenocarcinoma cell line, regarded as the “gold standard” in vitro model to study intestinal absorption [32], has been used in order to compare the results with our earlier findings [31]. By using MTT and LDH cytotoxicity assays, distinct treatment strategies—direct and simultaneous administration of cyanidin, its glycosylated derivative cyanidin-3-*O*-glucoside and quercetin—were assessed for their protective in vitro effects against the previously mentioned “non-essential” heavy metals. The antioxidant capacity of the extract was also determined by the ferric reducing antioxidant power (FRAP) and the bovine brain peroxidation assays.

## 2. Materials and Methods

### 2.1. Chemicals and Reagents

High glucose (4.5 g/L) Dulbecco’s modified Eagle medium (DMEM), fetal bovine serum ((FBS, PAN Biotech, Aidenbach, Germany), L-glutamine, trypsin (2.5% solution of 1:250 trypsin), MTT (3-(4,5-dimethylthiazol-2-yl)-2,5- diphenyltetrazolium bromide), LDH (lactate dehydrogenase), EDTA (ethylenediamimetetraacetic acid), CdCl_2_, HgCl_2_, or PbCl_2_, thiobarbituric acid (TBA), phosphate-buffered saline (PBS), bovine brain extract, FeCl_3_, ascorbic acid, butylated hydroxytoluene (BHT) and propyl gallate were purchased from Sigma-Aldrich S.p.a. (Milan, Italy). Quercetin was bought from Extrasynthese (Genay Cedex, France), and cyanidin and cyanidin-3-*O*-glucoside were bought from Phytolab GmbH & Co.KG (Vestenbergsgreuth, Germany). All solvents used for extraction and chemical analyses were of high purity and were obtained from VWR International s.r.l. (Milan, Italy).

### 2.2. Plant Material, Preparation of the Outer Layer Extract and Chemical Analysis

Tropea red onion (*A. cepa* L. var. Tropea) outer layer extract was prepared from the plant bulbs collected in Tropea (Calabria, Italy) in 2022. The samples were obtained from a local market (leg. det. Filomena Conforti). The dry outer layer was extracted by maceration at room temperature for 48 h × 3 times with 70% aqueous EtOH solution. Phenolics content and composition of the freshly made hydroalcoholic macerate were checked by thin layer chromatography (TLC), high-performance liquid Chromatography (HPLC)-diode array detection (DAD) and HPLC-high-resolution mass spectrometry (HRMS) following conventional procedures as previously described in Marrelli et al. [31] The analyses allowed us to confirm its composition as previous reported [30], with quercetin and some quercetin glucosides as the most abundant components and cyanidin 3-*O*-glucoside as a minor constituent.

### 2.3. Preparation of the Sample Solutions for the Bioassays

The extract prepared from Tropea red onion dry outer layer (5 mg/mL) was sterilized, filtrated (0.22 μm micro filters, Sartorius, Göttingen, Germany) and then used in the bioassays. A series of sequential dilutions were prepared to achieve the required final concentrations in each specific test with the extract. Stock solutions of each phenolic compound were prepared by stirring in distilled water to reach a final concentration of 5 mg/mL.

### 2.4. Determination of FRAP Activity

The ferric reducing potential of the plant extract was determined using the FRAP test [33] based on the reduction at low pH of a colorless ferric complex (Fe^3+^-tripyridyltriazine) to give a blue-colored ferrous complex (Fe^2+^-tripyridyltriazine) due to the presence of antioxidants in the sample. The change in absorbance, measured at 593 nm (Perkin Elmer Lambda 40 UV/VIS spectrophotometer, Milan, Italy), gives the extension of the reduction. The FRAP reagent was prepared by mixing 300 mM acetate buffer, pH 3.6, 10 mM TPTZ (2,4,6-tri(2-pyridyl)-s-triazine) in 40 mM HCl and 20 mM FeCl_3_ (10:1:1). A calibration curve was prepared with various concentrations of FeSO_4_. The reaction mixture was incubated in a water bath (37 °C) for 30 min. Data were calculated and expressed as μmol of Fe^2+^ *per* g of extract. All the measurements were performed in triplicate.

### 2.5. Bovine Brain Peroxidation Assay

A thiobarbituric acid (TBA) test measures the amount of free malondialdehyde (MDA), which is produced due to the peroxidation of membrane lipids [34]. TBA reacts with MDA to yield, in the acidic environment of reaction, a red adduct, which shows absorption at 532 nm and is readily extractable with organic solvents. The presence of antioxidant compounds in the reaction mixture used in the bovine brain peroxidation assay would reduce the extent of peroxidation. The TRO extract was checked for its antioxidant activity against liposomes made from a bovine brain extract (PBS, 5 mg/mL). FeCl_3_ (1 mM) and ascorbic acid (1 mM) were used to start the peroxidation, followed by incubation at 37 °C for 20 min. BHT in ethanol was added to prevent lipid peroxidation during the TBA test. Propyl gallate (0.1 mM) was used as a positive control. Five different solutions were prepared: 1 (liposomes alone), 2 (full reaction mixture with all the reagents), 3 (full reaction mixture with all the reagents plus positive control), 4 (full reaction mixture with all the reagents plus extract), 5 (extract alone). The full reaction mixture was prepared as follows: PBS (0.5 mL) was mixed with liposomes (0.2 mL), FeCl_3_ (0.1 mL), ascorbic acid (0.1 mL), BHT (0.1 mL), TBA 0.5 mL. Different concentrations of the extract were tested in order to calculate the IC_50_ value. The 50% inhibitory concentration (IC_50_) was calculated from the Prism dose–response curve (statistical program) obtained by plotting the percentage of inhibition versus the concentrations.

The percentage of Inhibition of lipid peroxidation for each concentration was calculated using the following formula:

% inhibition = [(FRM-B) − (ET-B-EA)/(FRM-B)] × 100

where FRM is the absorbance of the control reaction and ET is the absorbance in the presence of the sample. The absorbance of liposomes alone (B) and extract alone (EA) was also considered.

### 2.6. Preparation of “Non-Essential” Heavy Metals Solutions

Cd, Hg and Pb were given in the form of their water-soluble salts CdCl_2_, HgCl_2_, or PbCl_2_. A stock solution was made by dissolving 0.2283 g, 0.2715 g and 0.2781 g, respectively, of powdered CdCl_2_, HgCl_2_, or PbCl_2_ in 10 mL of bidistilled sterile water while stirring and then filtering the mixture. At the end, every stock solution had a concentration of 1 × 10^−1^ M [35]. The stock solution was diluted scalarly to create the CdCl_2_, HgCl_2_, or PbCl_2_ test solutions, which ranged in concentration from 0.01 to 250 μM. Before being used, they were all kept at 4 °C.

### 2.7. Culture Cells

The human Caco-2 adenocarcinoma cells were cultured in 25 cm^2^ flasks (Corning Inc., New York, NY, USA) with high glucose (4.5 g/L) Dulbecco’s modified Eagle’s medium (DMEM) supplemented with 4 mM L-glutamine ((Sigma-Aldrich S.p.a. Milan, Italy), 1% (*v*/*v*) antibiotic solution containing 100 U/mL penicillin (5%) and 100 mg/mL streptomycin (5%) and 10% (*v*/*v*) fetal bovine serum (FBS, PAN Biotech). Relative humidity of 95%, 5% CO_2_ and 37 °C were the conditions under which the cells were incubated (Thermo Scientific Hera Cell 240i, Waltham, MA, USA). A phosphate-buffered saline solution (PBS) was used to remove unattached cells from cultured cells 80% confluent; attached cells were harvested with 0.53 mM EDTA solution and 1 mL of 0.25% trypsin and then plated in 96-well microplates at the seeding density of 5000 cells/well. They were incubated for 24 h to allow cells adhesion before treatment with CdCl_2_, HgCl_2_, or PbCl_2_, TRO hydroalcoholic extract, cyanidin, cyanidin-3-*O*-glucoside and quercetin. In order to monitor the toxicity of CdCl_2_, HgCl_2_, or PbCl_2_, the extract and cyanidin, cyanidin-3-*O*-glucoside and quercetin alone or in combinations with “non-essential” heavy metals on Caco-2 cells, the following experimental sets were prepared: (1) cells were treated with increasing concentrations of CdCl_2_, HgCl_2_, or PbCl_2_ (0.01, 0.05,0.25, 2.5, 25 and 250 μM), plated in 6 wells/concentration group and cultured for 24 and 72 h; (2) cells were exposed to increasing concentrations (25, 50 and 100 μg/mL) of the extract, cyanidin, cyanidin-3-*O*-glucoside and quercetin, respectively, and incubated for 24 h and 72 h; (3) cells were treated with mixtures of CdCl_2_, HgCl_2_, or PbCl_2_ (25 μM)/TRO extract, cyanidin, cyanidin-3-*O*-glucoside and quercetin, respectively, at the concentrations of 25, 50 and 100 μg/mL and grown for 24 and 72 h. The TRO extract was only checked in combination with HgCl_2_ or PbCl_2_ (25 μM) at the same concentrations and incubation times as above. Untreated Caco-2 cells were processed in the same manner and incubated simultaneously to the treated groups.

### 2.8. Determination of Cell Viability

Cell viability was determined with the MTT test, which is based on the ability of mitochondrial oxidoreductases to convert soluble MTT into insoluble formazan in viable cells. The amount of formazan generated in the enzymatic reaction indicates the number of living cells [36]. In brief, Caco-2 cells were seeded in a 96-well plate at a density of 5 × 10^4^ and then incubated with increasing concentrations of CdCl_2_, HgCl_2_, or PbCl_2_ and Tropea red onion extract, cyanidin or cyanidin-3-*O*-glucoside, or quercetin, respectively (6 wells/concentration group plus 1 control group) for 24 and 72 h. Following this, the medium was taken out of the well and incubated for 3 h at 37 °C in the dark with 20 μL of the MTT stock solution (5 mg/mL in PBS 1X) in 180 μL of medium. A total of 150 μL of DMSO was added to dissolve the formazan crystals after the medium was removed, and the mixture was stirred at room temperature for five minutes. Finally, the absorbance was recorded at 540 nm with a multilabel microplate reader Victor 3 (PerkinElmer, Waltham, MA, USA). Each MTT assay was run in triplicate. The following equation was used to calculate the percentage of the control group (% control) that represented cell viability:

% control = Absorbance treatment/Absorbance control × 100%.

Data were expressed as the mean percentages of viable cells vs. the respective controls. Control groups consisted of cells which were processed in the same manner and incubated simultaneously to the treated groups.

### 2.9. Lactate Dehydrogenase (LDH) Assay

Lactate dehydrogenase (LDH) leakage into the culture medium was measured to assess cytotoxicity. Following the exposure to CdCl_2_, HgCl_2_, or PbCl_2_ and Tropea red onion extract, cyanidin, cyanidin-3-*O*-glucoside and quercetin, respectively, the recovered culture medium was centrifuged for 5 min at 3000 rpm to obtain a cell-free supernatant. This assay measures the capacity of LDH to oxidize lactate to pyruvate and generate NADH. The change in the absorbance determined by the enzymatic reaction was recorded at 440 nm with a microplate reader ((Bio-Rad-680, Bio-Rad, Redmond, WA, USA).

Cell LDH release (% control) was calculated with the following equation [37]:

% control = (U LDH/mg cell protein) treatment/(U LDH/mg cell protein) control × 100%.

### 2.10. Statistical Analysis

Data were expressed as mean ± SEM of three independent experiments. Data normality was evaluated using the D’Agostino–Pearson’s K2 test, and homogeneity of variances was estimated with the Levene’s test. The fitting procedure was carried out with the GraphPad Prism 9 (Graph Pad Software Inc., San Diego, CA, USA) statistical software package. One-way ANOVA and the Dunnett’s multiple comparison test (Sigma Stat Software 3.5, Systat Software Inc., San José, San Rafael, CA, USA) were utilized to define statistically significant differences between treated and control groups.

## 3. Results

### 3.1. Antioxidant Activity

In this study, the TRO extract, at a concentration of 2.5 mg/mL, showed a higher FRAP value (102.7 ± 4.8 μM Fe (II)/g raw material) than that reported for BHT (64.1 ± 3.6) used as positive control at the same concentration.

**Figure 1:**
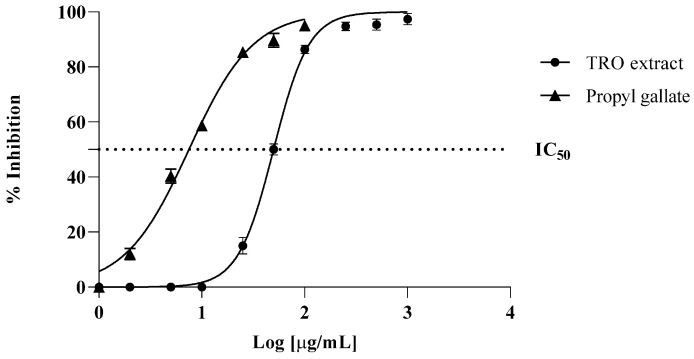
Inhibition (%) of liposomes lipid peroxidation by TRO extracts. Data are means ± S.D. of three determinations. Propyl gallate was the positive control.

As can be seen in Figure 1, the protective action of TRO against MDA production was strong, with an IC_50_ value of 50 μg/mL, although it was lower than the propyl gallate used as a positive control, which showed an IC_50_ value of 7 μg/mL. IC_50_ value (μg/mL) was also calculated by testing different concentrations of the extract. These results corroborate our FRAP results, also reported in this study, indicating that Tropea red onion outer scales are rich in antioxidant metabolites.

### 3.2. Cytotoxic Activity

The toxic effects of each of the “non-essential” heavy metals (Cd, Hg and Pb) included in our study were first evaluated by measurement of Caco-2 cell viability after 24 h and 72 h exposure to different concentrations of CdCl_2_, HgCl_2_, or PbCl_2_ (0.01–250 µM). In comparison with controls, a significant decrease in the cell viability of approximately 50% was observed after 24 h of exposure to the “non-essential” heavy metals at 25 µM concentrations (Table 1). After 72 h, a decrease in Caco-2 cell viability of about 50% was observed at lower concentrations of the “non-essential” heavy metals: namely, at 0.25 μM for the exposure to CdCl_2_, 2.5 μM to HgCl_2_ and 0.05 μM to PbCl_2_. Exposure to “non-essential” heavy metals reduced cell viability in a time dose-dependent manner (Table 1). Overall, the obtained results indicate that a 24 h treatment is sufficient to produce the “non-essential” heavy metals toxic effect on Caco-2 cells.

The potential effects of the TRO extract, cyanidin, cyanidin-3-*O*-glucoside and quercetin on Caco-2 cell viability were also investigated with the MTT assay. Cells were incubated with the different test samples at the increasing rates of 25, 50 and 100 μg/mL over 24 h and 72 h incubation. Compared to the positive control (cells treated only with the medium), cells treated with the extract, cyanidin, cyanidin-3-*O*-glucoside and quercetin displayed a different ability to affect Caco-2 cell viability.

**Figure 2:**
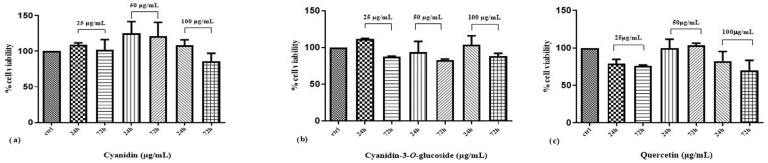
(**a**–**c**) Effect of cyanidin, cyanidin-3-*O*-glucoside and quercetin on Caco-2 cell viability after 24 h and 72 h of exposure (MTT assay). Data are expressed as a percentage of vehicle-treated cells (control). Results are expressed as mean ± SEM (*n* = 3). Non-significant differences are not shown.

The efficacy of cyanidin, cyanidin-3-*O*-glucoside and quercetin on Caco-2 to cell viability is shown in Figure 2. A concentration of 50 μg/mL of cyanidin produced a slight increase in cell viability at both 24 and 72 h treatments, while cell exposure to cyanidin-3-*O*-glucoside or quercetin did not affect cell viability at the times and concentrations used.

The effects on Caco-2 cell viability of mixtures of HgCl_2_ or PbCl_2_ with the extract were also evaluated. In these experiments, the TRO extract was used at the same concentrations as above, whereas for the “non-essential” heavy metals, the 25 μM concentration, which induced a significant cell viability decrease, was chosen. As a result, the 24 h treatment of Caco-2 cells with both combinations of HgCl_2_ or PbCl_2_ and the extract were effective in providing cytoprotection at all tested concentrations (25, 50 and 100 μg/mL). Conversely, the 72 h treatment resulted to be ineffective (Table 2).

**Figure 3:**
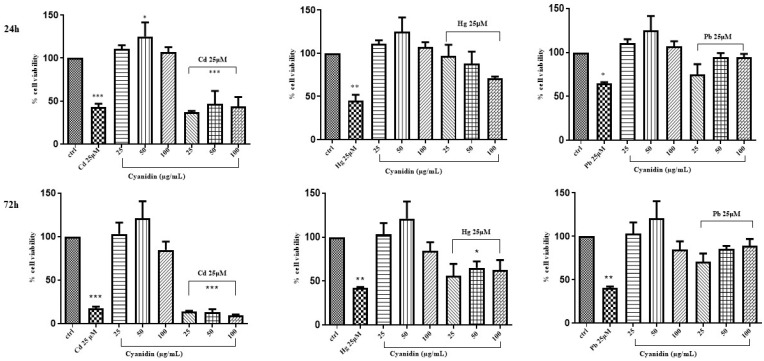
Effect of simultaneous treatment with CdCl_2_, HgCl_2_, or PbCl_2_ and cyanidin on Caco-2 cell viability after 24 h and 72 h of exposure (MTT assay). The mean ± SEM (*n* = 3) is used to express the data. Significant differences versus control * *p* < 0.05, ** *p* < 0.01, *** *p* < 0.001, **** *p* < 0.0001, whereas non-significant differences are not shown.

**Figure 4:**
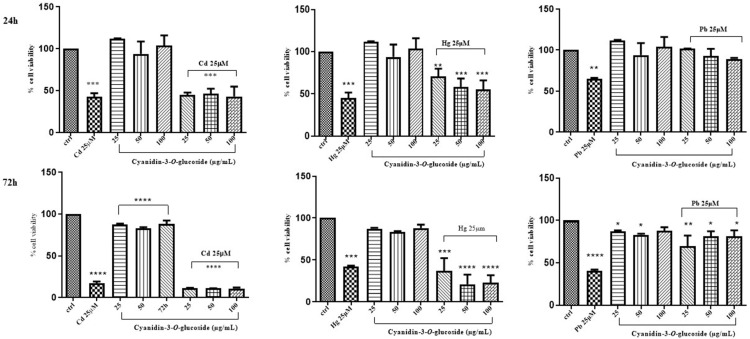
Effect on Caco-2 cell viability of the simultaneous exposure (24 h and 72 h) to CdCl_2_, HgCl_2_, or PbCl_2_ and cyanidin-3-*O*-glucoside (MTT assay). The mean ± SEM (*n* = 3) is used to express the data. Significant differences versus control * *p* < 0.05, ** *p* < 0.01, *** *p* < 0.001, **** *p* < 0.0001, whereas non-significant differences are not shown.

**Figure 5:**
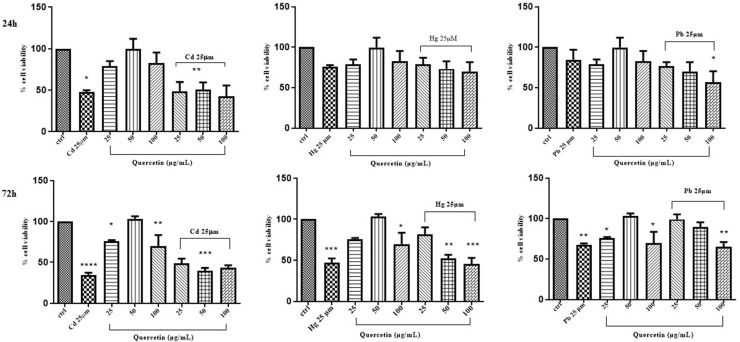
Effect on Caco-2 cell viability of the simultaneous exposure (24 h and 72 h) to CdCl_2_, HgCl_2_, or PbCl_2_ and quercetin (MTT assay). The mean ± SEM (*n* = 3) is used to express the data. Significant differences versus control * *p* < 0.05, ** *p* < 0.01, *** *p* < 0.001, **** *p* < 0.0001, whereas non-significant differences are not shown.

The same evaluation was performed in Caco-2 cells treated with mixtures of cyanidin, cyanidin-3-*O*-glucoside, or quercetin and CdCl_2_, HgCl_2_, or PbCl_2_. Cellular viability was assessed by incubating the cells with the above phytochemicals at the rates of 25, 50 and 100 μg/mL for 24 h and 72 h. Compared to the control, Caco-2 cell exposure to cyanidin, cyanidin-3-*O*-glucoside and quercetin did not afford any significant variations of cell viability at the used times and concentrations (Figure 3, Figure 4 and Figure 5).

### 3.3. Induction of Necrosis by “Non-Essential” Heavy Metals

To explore the ability of Cd, Hg and Pb to induce cell necrosis, the LDH leakage, a general hallmark of cell membrane damage and necrotic cell death, was measured. LDH leakage in Caco-2 cells treated with 25 μM of HgCl_2_ and PbCl_2_, respectively, was reduced by adding mixtures of TRO extract and HgCl_2_ and PbCl_2_ (50,100 μg/mL + 25 μM after 24 h); conversely, under the same experimental conditions, and after 72 h, LDH leakage resulted to be less significant (Table 3).

**Figure 6:**
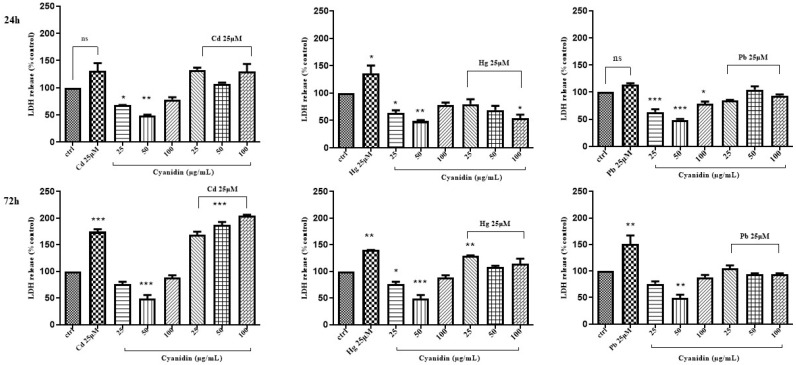
LDH activity of Caco-2 cells after 24 h and 72 h treatment with different concentrations of cyanidin and CdCl_2_, HgCl_2_, or PbCl_2_. The mean ± SEM (*n* = 3) is used to express the data. Significant differences versus control * *p* < 0.05, ** *p* < 0.01, *** *p* < 0.001, **** *p*< 0.0001, whereas non-significant differences are not shown.

**Figure 7:**
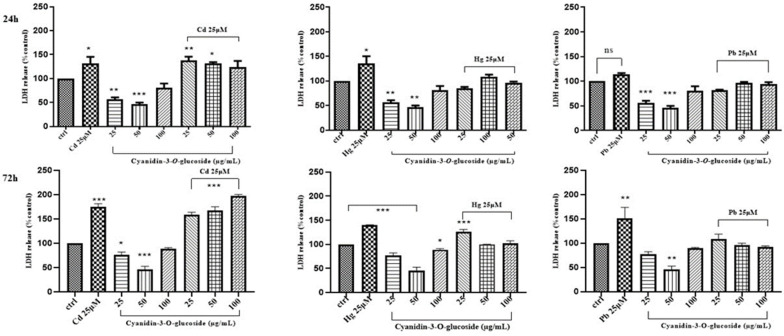
LDH activity of Caco-2 cells after 24 h and 72 h treatment with different concentrations of cyanidin-3-O-glucoside and CdCl_2_, HgCl_2_, or PbCl_2_. The mean ± SEM (*n* = 3) is used to express the data. Significant differences versus control * *p* < 0.05, ** *p* < 0.01, *** *p* < 0.001, **** *p* < 0.0001, whereas non-significant differences are not shown.

**Figure 8:**
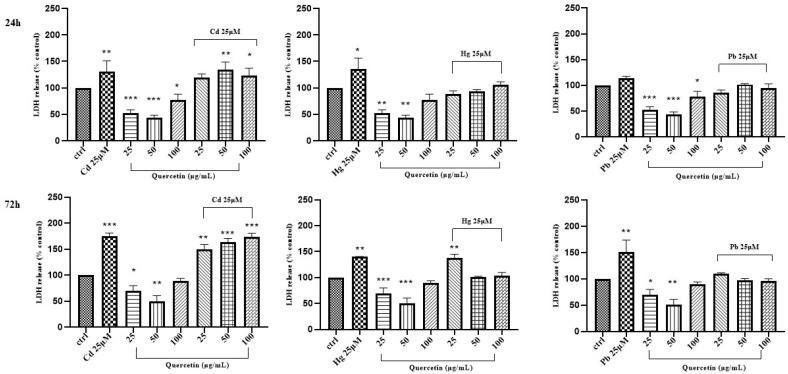
LDH activity of Caco-2 cells after 24 h and 72 h treatment with different concentrations of quercetin and CdCl_2_, HgCl_2_, or PbCl_2_. The mean ± SEM (*n* = 3) is used to express the data. Significant differences versus control * *p* < 0.05, ** *p* < 0.01, *** *p* < 0.001, **** *p* < 0.0001, whereas non-significant differences are not shown.

On the contrary, when combinations of the above “non-essential” heavy metals and the phytochemicals were used to treat Caco-2 cells, a dose/time-dependent reduction of LDH leakage was only observed for cyanidin with HgCl_2_ after 24 h of treatment (Figure 6, Figure 7 and Figure 8).

## 4. Discussion

“Non-essential” heavy metals are distinguished by their physical properties and are generally defined as a group of inorganic chemical elements with a high atomic mass and a specific density higher than 5 g/cm^3^; among them cadmium, mercury and lead are included [2]. They occur naturally in our environment, causing pollution and being able to produce harmful effects on human health. Cd, Hg and Pb are the most widespread “non-essential” heavy metals, which contaminate the environment, are not biodegradable and accumulate in living organisms, particularly in the human body, even in small amounts [38]. “Non-essential” heavy metals can cross cell membranes and affect biological systems, damaging cell membranes and cell organelles including nuclei, mitochondria, endoplasmic reticulum and lysosomes [39]. In addition, they may influence the activity of a wide range of enzymes involved in cellular metabolism, detoxification and damage repair [40,41]. Therefore, toxic effects of “non-essential” heavy metals can have severe consequences for the human body and human health. They can influence central nervous functions causing mental disorders [42], damage blood components and can damage liver, lungs, kidneys and other vital organs, promoting various pathological conditions [5,43].

It is known that oxidants are implicated in many important human pathologies; therefore, antioxidant compounds have gained importance in the prevention of oxidation-associated diseases/disorders. Due to the general capacity of Cd, Hg and Pb to deteriorate the oxidant/antioxidant cell balance, several authors have suggested that administration of various antioxidants can prevent and reduce the risks of Cd, Hg and Pb toxicity in the body [17]. Endogenous enzymes such as SOD, CAT and GP can eliminate ROS at the molecular level and chelate the metals, thus reversing their toxic effects [44]. However, non-enzymatic antioxidants are ingested daily through the diet [45]. Phytochemicals such as carotenoids, flavonoids, polyphenols and vitamins (vit B, vit C, vit E) are present in fruits, vegetables, nuts, cereals, meat and milk. These bioactive substances can function as metal chelators or scavengers for oxygen free radicals, which enables them to be employed as organic counteragents for the toxicity of Cd, Hg and Pb [46,47]. Due to their comparable antioxidant qualities to garlic, supplements including ginger and onion protected rats against the gonadotoxic and spermiotoxic effects of Cd as well as the developmental and renal toxicity of Pb [48]. Furthermore, oral tomato administration has been demonstrated to dramatically lower the rat liver accumulation of Cd, Pb and Hg [49].

Our previous investigation with the hydroalcoholic extract of Tropea red onion dry outer scales showed that this extract rich in phenolics had a significant cytoprotective effect against Cd in Caco-2 adenocarcinoma cells [31]. A dose-dependent increase in the cell’s viability was in fact observed after 24 h of exposure, while after 72 h of exposure to the “non-essential” heavy metals, a decrease from 50% to 80% was observed at 100 μg/mL and 50 μg/mL, respectively. A remarkable cytoprotection was especially detected when mixtures of CdCl_2_ and the TRO extract were incubated for 24 h at 50 μg/mL + 25 μM and 100 μg/mL + 25 μM and for 72 h at 25 μg/mL + 25 μM. Moreover, as reported previously by us, this finding was also corroborated by the measurement of LDH leakage in Caco-2 cells treated with CdCl_2_. LDH release appeared reduced in Caco-2 cells when they were treated with mixtures of the TRO hydroalcoholic extract and CdCl_2_ (100 μg/mL + 25 μM after 24 h). Similarly to CdCl_2_, HgCl_2_ and PbCl_2_ were able to decrease Caco-2 cells survival in a dose-dependent manner at doses in the range of 0.01–250 μM. After 24 h of exposure, Caco-2 cells viability decreased by approximately 50% at 0.25 μM for all “non-essential” heavy metals, and, after 72 h under the same experimental conditions, the ranking order of “non-essential” heavy metals toxicity on cell viability was PbCl_2_ > CdCl_2_ > HgCl_2_. As previously observed for CdCl_2_, in Caco-2 cells exposed to HgCl_2_ and PbCl_2_, a significant LDH leakage was detected at the same rates that produced a significant decrease in cell viability, suggesting that induction of necrosis was one of the major causes of the cell viability reduction.

The TRO extract that was used in our previous works [31] and was freshly prepared for this new study was rich in quercetin and some quercetin glycosylated compounds, with a small amount of the anthocyanin cyanidin-3-*O*-glucoside. The results obtained from Caco-2 cells treated with cyanidin, cyanidin-3-*O*-glucoside and quercetin showed that these phytochemicals were not able to increase cell viability as much as the TRO extract, even after 72 h of incubation. Furthermore, the consequence of the exposure of Caco-2 cells to mixtures of “non-essential” heavy metals/new TRO extract were highly effective at all concentrations (25, 50 and 100 μg/mL) after 24 h of treatment, while they only showed a discrete effect after 72 h. The same evaluation was performed with Caco-2 cells treated with mixtures of cyanidin, cyanidin-3-*O*-glucoside and quercetin and CdCl_2_, HgCl_2_ and PbCl_2_. Compared with the control, treatment with these polyphenolics did not alter cell viability at the times and concentrations used.

## 5. Conclusions

The Tropea red onion extract studied by us in this experimental work showed a good protective activity, though to a different extent, against Caco-2 cells exposed to HgCl_2_ and PbCl_2_. Its protective activity against the “non-essential” heavy metals resulted to be generally higher than that of the two pure components of the extract, quercetin and cyanidin-3-*O*-glucoside, tested alone, suggesting that the overall effect was possibly due to the combined activity of all the polyphenolic constituents present in the plant extract. Flavonols structures such as quercetin and its glucosylated derivatives as well as anthocyanins such as cyanidin and its conjugated glucoside are in fact consistent with their capacity to form metal complexes, thus avoiding the metal-mediated generation of free oxidants in biological systems. In addition to this ability, polyphenolic compounds are also able to bind/inactivate free radicals and reactive oxygen species. Consistently with the finding that TRO extract is rich in antioxidant compounds, we can assume that its higher cytotoxicity, compared to that of the individual assayed phytochemicals (cyanidin, cyanidin-3-*O*-glucoside and quercetin), may be derived by the combined antioxidant and chelating properties of all the molecules extracted from the outer scales of Tropea red onions.

In conclusion, the findings of this research underscore the significant potential of Tropea red onion extract as a protective agent against “non-essential” heavy metals. Through comprehensive analysis and experimentation, we have demonstrated its remarkable ability to mitigate the adverse effects of these contaminants. The unique properties of Tropea red onion extract, rich in polyphenolics, offer a promising avenue for further exploration in the field of environmental and nutritional sciences. By harnessing its natural antioxidant and chelating capabilities, we can envision its application in various industries, ranging from food and agriculture to environmental remediation. However, further studies are warranted to elucidate the underlying mechanisms and optimize its efficacy. Nevertheless, the results presented here provide compelling evidence of the protective role of Tropea red onion extract, paving the way for its potential integration into practical solutions aimed at safeguarding human health and environmental well-being.

## Figures and Tables

**Table 1:** Effect of CdCl_2_, HgCl_2_, or PbCl_2_ on Caco-2 cell viability after 24 h and 72 h of treatment.

Concentration	% Cell Viability	
	24 h	72 h
CdCl_2_		
0.01 µM	96.4 ± 1.5	52.9 ± 4.2 ***
0.05 µM	94.4 ± 5.0	58.7 ± 5.1 ***
0.25 µM	92.7 ± 2.5	44.9 ± 2.9 ***
2.5 µM	85.7 ± 3.0 *	23.3 ± 2.3 ***
25 µM	42.7 ± 3.0 ***	17.2 ± 1.5 ***
100 µM	23.8 ± 3.0 ***	14.7 ± 2.2 ***
250 µM	16.4 ± 2.5 ***	12.6 ± 2.6 ***
HgCl_2_		
0.01 µM	82.6 ± 2.5 *	62.4 ± 0.6 ***
0.05 µM	82.2 ± 2.2 *	60.0 ± 0.5 ***
0.25 µM	75.3 ± 1.7 **	58.9 ± 0.8 ***
2.5 µM	62.2 ± 3.8 ***	52.1 ± 1.8 ***
25 µM	45.1 ± 1.7 ***	42.4 ± 0.6 ***
100 µM	23.9 ± 1.8 ***	21.0 ± 0.2 ***
250 µM	19.1 ± 1.2 ***	18.8 ± 0.8 ***
PbCl_2_		
0.01 µM	81.7 ± 2.6 *	64.1 ± 1.2 ***
0.05 µM	80.6 ± 2.5 *	47.3 ± 1.0 ***
0.25 µM	75.0 ± 2.4 **	46.2 ± 0.6 ***
2.5 µM	71.8 ± 2.5 **	41.0 ± 0.7 ***
25 µM	64.7 ± 1.6 ***	40.6 ± 1.0 ***
100 µM	66.1 ± 1.2 ***	28.5 ± 1.1 ***
250 µM	25.9 ± 1.1 ***	24.6 ± 1.0 ***
Ctrl	100 ± 0.0	100 ± 0.0

Data are expressed as a percentage of vehicle-treated cells (control). Results are shown as mean ± SEM (*n* = 3). * *p* < 0.05, ** *p* < 0.01, *** *p* < 0.001, **** *p* < 0.0001 as compared with the control.

**Table 2:** Effect of TRO extract and simultaneous treatment with CdCl_2_, HgCl_2_, or PbCl_2_ and TRO extract on Caco-2 cell viability after 24 h and 72 h of exposure (MTT assay).

Concentration	% Cell Viability			
	24 h		72 h	
Cd 25 µM	45.8 ± 3.5		14.4 ± 1.5	
	TRO	TRO + CdCl_2_	TRO	TRO + CdCl_2_
25 µg/mL	143.9 ± 13.9	39.4 ± 3.8 *	110.2 ± 8.4	103.3 ± 18.2
50 µg/mL	203.9 ± 19.7 ***	189.7 ± 18.3 **	17.3 ± 1.1 ***	57.6 ± 2.9 **
100 µg/mL	207.2 ± 22.6 ***	142.9 ± 17.9	54.3 ± 1.5 **	16.3 ± 9.1 ***
Hg 25 µM		44.5 ± 1.7		34.6 ± 4.3
	TRO	TRO + HgCl_2_	TRO	TRO + HgCl_2_
25 µg/mL	143.9 ± 13.9 **	183.6 ± 5.3 **	110.2 ± 8.4	54.3 ± 2.0
50 µg/mL	203.9 ± 19.7 ***	192.3 ± 6.0 ***	17.3 ± 1.1 **	57.6 ± 2.9
100 µg/mL	207.2 ± 22.6 ***	228.4 ± 9.9 ***	54.3 ± 1.5 *	74.3 ± 2.0
Pb 25 µM		64.3 ± 3.9		42.1 ± 0.7
	TRO	TRO + PbCl_2_	TRO	TRO + PbCl_2_
25 µg/mL	143.9 ± 13.9	138.7 ± 8.9	110.2 ± 8.4	95.6 ± 6.5
50 µg/mL	203.9 ± 19.7 **	166.4 ± 12.2 *	17.3 ± 1.1 **	78.7 ± 17.5
100 µg/mL	207.2 ± 22.6 **	203.4 ± 6.5 ***	54.3 ± 1.5 *	103.3 ± 18.2
Ctrl	100		100	

Data are expressed as mean ± SEM (*n* = 3). Significant differences versus control * *p* < 0.05, ** *p* < 0.01, *** *p* < 0.001, **** *p* < 0.0001.

**Table 3:** LDH activity of Caco-2 cells after 24 h and 72 h treatment with different concentrations of TRO extract and CdCl_2_, HgCl_2_, or PbCl_2_.

Concentration	% Cell Viability			
	24 h			
25 µM Metal		Cd 144.4 ± 14.8	Hg 135.4 ± 14.9	Pb 126.6 ± 2.8
	TRO	TRO + CdCl_2_	TRO + HgCl_2_	TRO + PbCl_2_
25 µg/mL	105.5 ± 5.0 **	93.4 ± 5.3	113.0 ± 6.8	114.6 ± 5.5
50 µg/mL	66.7 ± 9.9 ***	55.5 ± 7.4 ***	50.3 ± 7.8 ***	65.3 ± 0.7 ***
100 µg/mL	85.0 ± 2.4 **	46.7 ± 4.0 ***	30.3 ± 5.5 ***	27.6 ± 3.0 ***
	72 h			
25 µM Metal		Cd 149.3 ± 10.0	Hg 144.4 ± 6.9	Pb 160.9 ± 8.0
	TRO	TRO + CdCl_2_	TRO + HgCl_2_	TRO + PbCl_2_
25 µg/mL	100.5 ± 10.0 **	98.7 ± 6.0 ***	106.5 ± 5.1	102.5 ± 8.0
50 µg/mL	110.1 ± 9.5 **	96.9 ± 6.0 ***	149.2 ± 4.9	53.7 ± 4.3 ***
100 µg/mL	96.6 ± 6.0 ***	134.7 ± 12.0	158.9 ± 0.5	70.0 ± 2.0 ***
Ctrl	100		100	

The mean ± SEM (*n* = 3) is used to express the data. Significant differences versus control * *p* < 0.05, ** *p* < 0.01, *** *p* < 0.001, **** *p* < 0.0001.

## Data Availability

The data presented in this study are available in the article.
